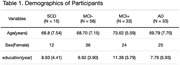# Different functional structures of the brainstem according to dementia subtypes

**DOI:** 10.1002/alz.092088

**Published:** 2025-01-09

**Authors:** Kim Seul Gi

**Affiliations:** ^1^ Ajou University School of Medicine, Korea, Suwon Korea, Republic of (South)

## Abstract

**Background:**

The extent of neurofibrillary changes, one of the pathological hallmarks of AD, correlates with the severity of AD in dementia. The brainstem is known to be the site of neurofibrillary changes in the early stages of Alzhimer’s disease. The neurotransmitter system in the brainstem processes information from subcortical and cortical circuits affect to various cognitive and behavioral responses in the cerebral cortex. This study aims to investigate we cluster the brainstem with respect to how they connect to the cortex and focus on the brainstem cluster of differences according to the subtypes of AD.

**Method:**

We acquired the resting state fMRI data from 137 patients with several dementia types (96 females, mean age = 70.31 ± 7.13), which were collected from the BICWALZS (Biobank Innovation for chronic Cerebrovascular disease With ALZheimer's disease Study). They were divided into four dementia subtype groups: subjective cognitive decline(SCD) mild cognitive impairment(MCI)‐β‐amyloid negative(MCI‐), MCI‐β‐amyloid positive (MCI+), and AD (N=33). For physiological denoising in functional neuroimaging data of the brainstem, we used FSL's ICA‐AROMA, a data‐driven denoising approach. We extracted each regional‐mean time series using Schaefer 200 atlas and voxel‐wise time series of brainstem. A functional connectivity matrix is composed of the brainstem‐to‐cortex that regressed out dominant pattern, which is the weights of the connectivity across all 200 cortical regions that the brainstem had. Then we clustered the brainstem based on modularity according to the extent of connectivity similarity between the brainstem and cortex via connectivity matrix profiles. Finally, our study focused that comparison the pattern of clustering according to the subtypes of AD.

**Results:**

We found that each community in the brainstem is a group with a similar pattern of functional connectivity to the cortex. Different brainstem communities are observed in the distinct in accordance with the subtypes of AD, suggesting that in each disease group can characterize specific brainstem communities with functional connectivity to the cerebral cortex.

**Conclusion:**

Different patterns of clusters in different subgroups of dementia may explain functional abnormalities in the brainstem as dementia progresses. The future study will characterize each connection between clusters of brainstem and cortical regions